# Using the AKAR3-EV biosensor to assess Sch9p- and PKA-signalling in budding yeast

**DOI:** 10.1093/femsyr/foad029

**Published:** 2023-05-12

**Authors:** Dennis Botman, Sineka Kanagasabapathi, Philipp Savakis, Bas Teusink

**Affiliations:** Systems Biology Lab, AIMMS/A-LIFE, Vrije Universiteit Amsterdam, 1081 HV Amsterdam, The Netherlands; Systems Biology Lab, AIMMS/A-LIFE, Vrije Universiteit Amsterdam, 1081 HV Amsterdam, The Netherlands; Systems Biology Lab, AIMMS/A-LIFE, Vrije Universiteit Amsterdam, 1081 HV Amsterdam, The Netherlands; Systems Biology Lab, AIMMS/A-LIFE, Vrije Universiteit Amsterdam, 1081 HV Amsterdam, The Netherlands

**Keywords:** *Saccharomyces cerevisiae*, FRET biosensor, intracellular signalling, PKA, Sch9, single-cell

## Abstract

Budding yeast uses the TORC1-Sch9p and cAMP-PKA signalling pathways to regulate adaptations to changing nutrient environments. Dynamic and single-cell measurements of the activity of these cascades will improve our understanding of the cellular adaptation of yeast. Here, we employed the AKAR3-EV biosensor developed for mammalian cells to measure the cellular phosphorylation status determined by Sch9p and PKA activity in budding yeast. Using various mutant strains and inhibitors, we show that AKAR3-EV measures the Sch9p- and PKA-dependent phosphorylation status in intact yeast cells. At the single-cell level, we found that the phosphorylation responses are homogenous for glucose, sucrose, and fructose, but heterogeneous for mannose. Cells that start to grow after a transition to mannose correspond to higher normalized Förster resonance energy transfer (FRET) levels, in line with the involvement of Sch9p and PKA pathways to stimulate growth-related processes. The Sch9p and PKA pathways have a relatively high affinity for glucose (K_0.5_ of 0.24 mM) under glucose-derepressed conditions. Lastly, steady-state FRET levels of AKAR3-EV seem to be independent of growth rates, suggesting that Sch9p- and PKA-dependent phosphorylation activities are transient responses to nutrient transitions. We believe that the AKAR3-EV sensor is an excellent addition to the biosensor arsenal for illuminating cellular adaptation in single yeast cells.

## Teaser

The AKAR3-EV biosensor measures the signalling activity of two major kinases -PKA and Sch9- in single living budding yeast cells.

## Introduction

One universal aspect of life is change, and the ability to adapt to it is a major determinant of reproductive success. For the unicellular organism *Saccharomyces cerevisiae* (budding or baker’s yeast), there is no exception. In the wild, this yeast lives on fruit and tree bark, where it endures feast-famine cycles (Liti 2015, Jouhten et al. 2016). In industry, domesticated yeast also experiences changing conditions when used in large-scale fermenters with inoculation transitions and poor stirring (Lara et al. 2006, Wehrs et al. 2019). For budding yeast, nutrient availability is a major environmental parameter that sets the investment in metabolism, stress resistance, and proliferation (Broach 2012, Conrad et al. 2014). Understanding the logic of these circuitries is a key challenge in cell biology. Also for industry, control over this response, e.g. by selection of certain preferred subpopulation or removal of undesired populations, could increase production efficiencies (Xiao et al. 2016). Nutrient adaptations can be captured on population level using bulk assays, but these methods may mask the true responses produced by the intracellular signalling circuits inside single cells. Thus, a more profound characterization of the cellular adaptation of budding yeast to nutrient changes at the single-cell level is highly desired.

Regarding carbon sources, fruit sugars are preferred by yeast, and therefore it has developed various pathways to sense and adapt to changes in the availability of these substrates (Rolland et al. 2002, Santangelo 2006, Rødkaer and Faergeman 2014). The cAMP-PKA pathway is one of the major signalling cascades that get activated when cells encounter fermentable sugars (Eraso and Gancedo 1985, Beullens et al. 1988, Botman et al. 2021). cAMP production is activated via two routes (Casperson et al. 1985, Kataoka et al. 1985, Broek et al. 1987, Beullens et al. 1988, Mbonyi et al. 1988, Munder and Küntzel 1989, Engelberg et al. 1990, van Aelst et al. 1990, van Aelst et al. 1991, Pardo et al. 1993, Colombo et al. 1998, Yun et al. 1998, Kraakman et al. 1999, Rolland et al. 2000, Rolland et al. 2001, Lemaire et al. 2004, Kim et al. 2013): via import and metabolism of sugars and via extracellular sensing of glucose and sucrose by the G-protein coupled receptor Gpr1p. These two inputs give a transient increase in cAMP, which causes the dissociation of Bcy1p (a PKA regulator) from the PKA subunits Tpk1-3. This finally results in an increase of PKA activity; PKA is a major effector kinase in yeast, accounting for 75%–90% of the cellular changes during a transition from a glucose-derepressed (respiratory) state to a fermentable glucose-repressed (fermentative) state (Thevelein 1994, Rolland et al. 2002, Winderickx et al. 2003, Santangelo 2006, Zaman et al. 2009, Broach 2012, Kunkel et al. 2019). The evoked transition gives a radical change in yeast physiology; cells change their metabolism from respiratory to fully fermentative, repress metabolic pathways for other carbon sources, decrease their stress resistance, and make large investments in ribosomal biogenesis.

The TORC1-Sch9 cascade is a second major signalling cascade in yeast cells (Crauwels et al. 1997, Roosen et al. 2004, Slattery et al. 2008, van Zeebroeck et al. 2021). In contrast to PKA, which gets activated by mostly fermentable sugars, Sch9p is activated by TORC1p when a complete palette of nutrients for growth (such as amino acids, nitrogen, phosphate, and a fermentable carbon source) is available (Crauwels et al. 1997, Conrad et al. 2017, van Zeebroeck et al. 2021). Although the two pathways can operate independently (Zurita-Martinez and Cardenas 2005), they have many positive interactions and can rescue each other’s activities, probably via the large overlap in their targets: Sch9 and PKA proteins both phosphorylate the RRxT motif (Reinders et al. 1998, Ma et al. 1999, Plank et al. 2020, Plank 2022). Activation of Sch9p activates 90% of the genes that PKA also activates (Zaman et al. 2009), making Sch9p as important as PKA for proper cellular decision making in yeast.

Currently, PKA and Sch9p activities are difficult to measure in (single) cells: the most common method is measuring the activity of trehalase, a PKA and Sch9p target, or using kemptide as a substrate (Crauwels et al. 1997, Roosen et al. 2004). However, these bulk assays lack single-cell information and show only static activity levels of PKA and Sch9p activity. Studies suggest that the PKA activity in yeast is more a transient phenomenon activated during (mostly) sugar transitions and that the TORC1-Sch9p axis dictates the steady-state growth mode of a yeast cell (Crauwels et al. 1997, Kunkel et al. 2019). Dynamic readouts are needed to substantiate these interesting hypotheses. Moreover, single-cell dynamics allows to test for heterogeneity during nutrient transitions—a phenomenon that we did not find for cAMP dynamics (Botman et al. 2021), but it is unknown if heterogeneity exists more downstream. Furthermore, the sensitivity of Sch9p and PKA activity with respect to glucose remains to be characterized. Finally, the basal RRxT phosphorylation status of the cell at various growth rates is also poorly characterized.

Here, we implemented and tested the mammalian PKA sensor AKAR3-EV in yeast to provide a tool that can help to enlarge our understanding of PKA and Sch9 signalling. The sensor allowed us to measure the single-cell dynamics of the PKA and Sch9p phosphorylation status in a robust and accurate manner. We found large heterogeneous responses of yeast cells for some nutrient transitions, which we did not previously find in cAMP dynamics. The detected heterogeneity potentially affects the overall cellular state since these two kinases constitute the vast majority of the cellular transition during a transition to a fermentable carbon source. Furthermore, our data implies that the phosphorylation status is not related to growth rate. How the two kinases regulate the cellular transitions at a single-cell level can be studied in more depth using this sensor.

## Material and methods

### Sensor construction

AKAR3EV with YPET-eCFP as a FRET pair was kindly provided by Dr. Aoki (Komatsu et al. 2011). The sensor was amplified using KOD One™ PCR Master Mix (Toyobo, Osaka, Japan) with 5′-ATGCTAGCACGGAGCTCACTGAATTCGGCATGGTGAG-3′ and 5′-ATGGATCCACGGTCGACACTTTAATCCAGAGTCAGGCG-3′ as forward and reverse primers, respectively. Next, the PCR product and the pDRF1-GW plasmid were digested using BamHI-HF and NheI-HF (New England Biolabs, Ipswich, MA, USA), and the PCR product was ligated into the plasmid using T4 DNA ligase (New England Biolabs), which yield pDRF1-GW AKAR3-EV, containing the AKAR3-EV sensor under PMA1 promotor expression.

AKAR3-EV-NR was constructed by performing a PCR with KOD One™ PCR Master Mix on pDRF1-GW AKAR3-EV with 5′- TATTCCGGATTGAGGCGCGCGGCCCTGGTTGACGGCGGCCGCATGGTGAGCAAGGGC-3′ as a forward primer and 5′-ATGGATCCACGGTCGACACTTTAATCCAGAGTCAGGCG-3′ as a reverse primer. The PCR product and pDRF1-GW AKAR3-EV were digested using Kpn2I and XbaI (Thermo Fisher Scientific, Waltham, MA, USA). Afterwards, the PCR product was ligated in the digested pDRF1-GW AKAR3-EV, replacing the sensor domain and eCFP with the non-responding (T506A) sensor domain and eCFP.

pDRF1-GW eCFP was made by performing a PCR with KOD One™ PCR Master Mix on AKAR3-EV with FW primer 5′-ATGCTAGCATGGTGAGCAAGGGCG-3′ and RV primer 5′- TAGCGGCCGCTTACTTGTACAGCTCGTCCATGCCG -3′, after which the PCR product and pDRF1-GW were digested using NheI-HF and NotI-HF (New England Biolabs). Finally, the PCR product was ligated into pDRF1-GW using T4 DNA ligase.

### Yeast transformation

Strains used in this study are listed in Table 1. Strains were transformed by resuspending yeast cells from either a YPAD plate or a selective plate in a transformation mixture containing 240 µL PEG 3350 (50% w/v), 40 µL 1 M LiAC, 10 µL Salmon Sperm DNA (10 mg/mL, Sigma–Aldrich, Stl. Louis, MO, USA), and 500–1000 ng plasmid DNA. Next, cells were incubated for 15–20 minutes at 42°C. Afterwards, the cells were centrifuged at 13 000 g for 30 seconds, the supernatant was removed, and 150 µL water was added. Finally, the cells were plated on selective plates.

**Table 1.** Used strains in this study.

Strain	Characteristics	Source
W303-1A WT	MATa, leu2-3112, trp1-1, can1-100, ura3-1, ade2-1, his3-11,15	In-house
W303-1A + pDRF1-GW	W303-1A MATa + pDRF1-GW	In-house
W303-1A + pYX212 AKAR3	W303-1A MATa + pYX212 AKAR3	This work, pYX212 AKAR3 was provided by Sonia Colombo (Colombo et al. 2017, Colombo et al. 2022)
W303-1A + pDRF1-GW AKAR3-EV	W303-1A MATa + pDRF1-GW AKAR3-EV	This work
W303-1A + pDRF1-GW AKAR3-EV-NR	W303-1A MATa + pDRF1-GW AKAR3-EV-NR	This work
W303-1A + pDRF1-GW *eCFP*	W303-1A MATa + pDRF1-GW eCFP	This work
W303-1A + pYES2_ACT1_ pHluorin	W303-1A MATa + pYES2_ACT1_ pHluorin	In-house, pYES2 pHluorin was provided by Gertien Smits (Orij et al. 2009, Orij et al. 2012)
W303-1A *CYR1^K1876M^*	W303-1A MATa + *CYR1^K1876M^*	(Vanhalewyn et al. 1999)
W303-1A *CYR1^K1876M^* + pDRF1-GW AKAR3-EV	W303-1A MATa *CYR1^K1876M^* + pDRF1-GW AKAR3-EV	This work
W303-1A *sch9Δ* + pDRF1-GW AKAR3EV	W303-1A MATa s*ch9::TRP1* + pDRF1-GW AKAR3EV	(Roosen et al. 2004)
W303-1A *sch9Δ*	W303-1A MATa s*ch9::TRP1*	In-house
W303-1A *ypk1Δ*	W303-1A *ypk1::TRP1*	(Niles and Powers 2014)
W303-1A *ypk1Δ* + pDRF1-GW AKAR3-EV	W303-1A *ypk1::TRP1* + pDRF1-GW AKAR3-EV	This work
SP1 WT	Matα his3, leu2, ura3, trp1, ade8, can1	(Ma et al. 1999)
S18-1D (SP1 *TPK1^wimp^*)	SP1 Matα *TPK1^wimp^, tpk2::HIS3, tpk3::TRP1*	(Nikawa et al. 1987)
SP1 + pDRF1-GW AKAR3EV	SP1 Matα + pDRF1-GW AKAR3EV	This work
SP1 + pDRF1-GW AKAR3	SP1 Matα + pYX212 AKAR3	This work, pYX212 AKAR3 was provided by Sonia Colombo (Colombo et al. 2017, Colombo et al. 2022)
SP1 TPK1^wimp^ + pDRF1-GW AKAR3EV	S18-1D Matα + pDRF1-GW AKAR3EV	This work
SP1 + pDRF1-GW AKAR3EV-NR	SP1 Matα + pDRF1-GW AKAR3EV-NR	This work
S25-31C	MATa his3, leu2, ura3, trp1, ade8, *tpk2::HIS3, tpk3::TRP1, bcy1::LEU2, Sch9::ADE8*	(Toda et al. 1988, Crauwels et al. 1997)
S25-31C + pDRF1-GW AKAR3EV	MATa his3, leu2, ura3, trp1, ade8, *tpk2::HIS3, tpk3::TRP1, bcy1::LEU2, Sch9::ADE8* + pDRF1-GW AKAR3EV	This work

### Yeast growth

Cells expressing URA3 plasmids (pDRF1-GW, pYES2, or pYX212) were grown overnight in 1x YNB medium (Sigma–Aldrich, St. Louis, MO, USA), containing 1% ethanol (Boom BV, Meppel, The Netherlands), 20 mg/L adenine hemisulfate (Sigma–Aldrich), 20 mg/L L-tryptophan (Sigma–Aldrich), 20 mg/L L-histidine (Sigma–Aldrich), and 60 mg/L L-leucine (SERVA Electrophoresis GmbH, Heidelberg, Germany). For WT strains, uracil (Sigma–Aldrich) was added to a final concentration of 20 mg/L. The cells were subsequently diluted and grown overnight to an OD_600_ of 0.1–1.5, with at least 5 divisions.

For the experiments that involved S25-31C, cells were grown on 1x YNB medium containing 1% ethanol, 5 mM glucose (Boom BV, Meppel, The Netherlands), 20 mg/L adenine hemisulfate, 20 mg/L L-tryptophan, 20 mg/L L-histidine, and 60 mg/L L-leucine until glucose was exhausted. Next, cells were kept on this medium for 2 more days, after which they were visualized under the microscope.

### Concanavalin (ConA) coverslips

ConA coverslips were made as described by Hansen et al. (2015). To prepare the coverslips, the ConA was diluted to 200 µg/mL and put on coverslips. The coverslips were dried overnight in a 6-well plate.

### Microscopy

Cells were grown as described and transferred to the 6-well plates containing the ConA-coated coverslips. Next, the coverslip was placed in a Attofluor cell chamber (ThermoFisher Scientific, Waltham, MA, USA), and 1 mL of medium was added to the cell chamber. The coverslips were visualized using a Nikon Ti-eclipse microscope (Nikon, Minato, Tokio, Japan) at 30°C equipped with a TuCam system (Andor, Belfast, Northern Ireland, UK) and 2 Andor Zyla 5.5 sCMOS Cameras (Andor) and a SOLA 6-LCR-SB light source (Lumencor, Beaverton, OR, USA). Cells expressing the sensors, except pHluorin, were excited via a 438/24 nm excitation filter (Semrock, Lake Forest, IL, USA), and emission was passed through a 458 nm long-pass (LP) dichroic mirror. The acceptor and donor emissions were filtered by a 542/27 and 483/32 nm filter (Semrock). Direct acceptor fluorescence was recorded with a 500/24 nm excitation filter, a 520 LP dichroic filter, and a 542/27 nm emission filter. pHluorin was excited at 460–500 and 380–420 nm, and emission was recorded at 510–560 nm. For all perturbations, a baseline was recorded, after which YNB medium containing the compound of choice in a 10x concentration was added and diluted in the cell chamber to a 1x concentration.

### Rapamycin experiments

Cells were grown as described and incubated with 200 nM rapamycin or a solvent (100% ethanol) for at least 2 h, after which the perturbations (addition of 10 mM glucose) were performed.

### Microscopy analysis

Cells were segmented using an in-house script. In brief, this script stabilizes any drift using the image stabilizer plugin (Li 2008). Next, background correction was performed, and cells were segmented using the Weka Segmentation plugin (Arganda-Carreras et al. 2017b, Arganda-Carreras et al. 2017a), and the mean fluorescence for each cell was calculated for each frame. The resultant text files were analysed using R 4.1.3. For all cells, 40% bleedtrough correction was performed and the FRET ratio (i.e. bleedtrough-corrected YFP divided by the CFP signal) was calculated. Finally, baseline normalization was performed for time-lapse data. pHluorin ratios were calculated by dividing the fluorescence at 380–420 nm excitation over the fluorescence at 460–500 nm.

For the dose-response fit, the final FRET levels (i.e. the mean FRET value of the last 3 frames) after the glucose additions were fitted (using the nls function in R) against the final glucose concentration according to equation 1, with [glucose] as the glucose concentration in mM, max as the maximal change in normalized FRET, and K_0.5_ as the glucose concentration giving 50% of the maximal response.


(1) \begin{eqnarray*} {\rm{normalized\,\, FRET\,\, response}} - 1{\rm{\ }} = \frac{{\max {\rm{\ }} \cdot [{\rm{glucose}}]}}{{{{\rm{K}}}_{0.5} + \left[ {{\rm{glucose}}} \right]}}\ \end{eqnarray*}

Clustering was performed using the factoextra package in R. The optimal amount of clusters was determined by eye.

For the long-term ethanol to mannose transition, microscopy images were segmented with a convolutional neural network with customized weights (Pachitariu and Stringer 2022). Frame-to-frame association of segmented objects was done through maximum matching based on inverse centroid distance. Fluorescence background was estimated from fluorescence images masked with the dilated segmentation images using the Background2D class from the photutils python package (Bradley et al. 2022). FRET ratios were calculated as the bleedthrough-corrected YFP signal, divided by the CFP signal. For growth type classification, cells that were in or that reached G2-phase (i.e. cells that had or did get a bud) during the experiment (between 2 and 6 h after the transition) were manually identified and classified as growing if there was a perceptible increase in bud volume, and as non-growing if there was no such perceptible increase. Analysis was restricted to cells that were present at the start of the experiment.

### Flow cytometry

Cells were grown as described with YNB medium containing either 1% ethanol, 100 mM glucose, 100 mM galactose, or 100 mM mannose. Next, samples were measured using a CytoFLEX S Flow Cytometer (Beckman Coulter, Brea, CA, USA). Cells were excited using a 405 and a 488 nm laser, and emission fluorescence was passed through a 470/20 and 525/40 nm filter and recorded by avalanche photodiodes. Events with a saturating forward or side scatter were filtered, after which the median fluorescence signal of the cells expressing the empty pDRF1-GW plasmid was subtracted from all samples. Next, bleedtrough was calculated using the eCFP-expressing strain, and cells with at least a acceptor fluorescence signal of 2500 (arbitrary units) were kept. Lastly, FRET ratios were calculated for all remaining cells.

### Growth assays

Yeast strains were grown on 1% ethanol medium as described. Cells were washed twice by centrifuging at 3500 g for 3 minutes and resuspending in YNB medium without a carbon source. Afterwards, cells were resuspended in YNB medium without carbon source to an OD_600_ of 1. Next, 20 μL of cells were put in a well of a 48-well microtiter plate having 480 μL YNB medium containing either 10 mM glucose, 10 mM galactose, or 0.1% ethanol. OD_600_ was measured every 5 minutes with a CLARIOstar plate reader (BMG LABTECH, Ortenberg, Germany) at 30°C and 700 rpm orbital shaking. Growth rates were calculated by calculating a moving average over each growth curve. Next, a sliding window was used in which a linear regression was fitted for each window, which gives the growth rate during this window. Next, to reduce the effect of outlier growth rates of the sliding window fits, the tenth fastest found slope was selected as the determined growth rate.

## Results

### AKAR3-EV shows a FRET response in budding yeast.

To visualize the cellular kinase activities of Sch9p and PKA, we tested the use of the AKAR3-EV sensor, which consists of YPET-FHA1-EVlinker-RRAT_motif_-eCFP (Zhang et al. 2001, Allen and Zhang 2006, Komatsu et al. 2011). This sensor, developed for mammalian PKA assays, should also work in yeast as PKA and Sch9p also have RRxT as recognition site (Reinders et al. 1998, Ma et al. 1999, Plank et al. 2020). We constructed a non-responsive AKAR3-EV-NR sensor as a control by mutating the threonine in the RRxT motif to alanine (T506A), and expressed both AKAR3-EV and AKAR3-EV-NR in the W303-1A and SP1 strains. Both W303-1A and SP1 were used as some mutant W303-1A strains have difficulties to express sensors. We previously also observed this for the yEPAC cAMP sensor or even single fluorescent proteins, which indicates this is a more general problem for some W303-1A mutants and not a sensor-specific issue.

**Figure 1.**
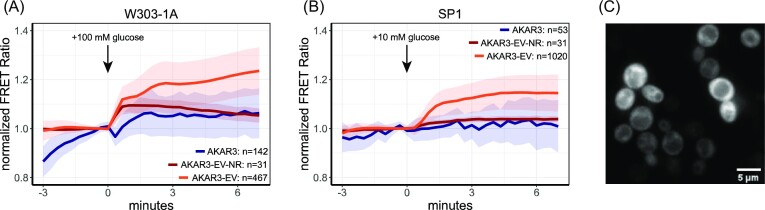
AKAR3-EV responds to glucose addition. (A) Response of W303-1A WT cells expressing AKAR3, AKAR3-EV NR, or AKAR3-EV grown on 1% ethanol to 100 mM glucose addition. (B) Response of SP1 WT cells expressing either AKAR3, AKAR3-EV NR, or AKAR3-EV grown on 1% ethanol to 10 mM glucose addition. (C) Expression and distribution of W303-1A cells expressing AKAR3EV. Lines show the mean FRET value, normalized to the baseline, shades indicate SD, colours indicate the expressed sensor. All data are obtained from at least two biological replicates.

In these two strains, we assessed the FRET response from a 1% ethanol to a 100 or a 10 mM glucose transition (Figs. 1A, B, and Supplementary Fig. S1). Furthermore, we tested the older generation AKAR3 sensor for performance comparison (Colombo et al. 2017, Colombo et al. 2022). The AKAR3-EV sensor gave a clear increase in FRET after glucose addition, which was 3–4 times higher compared to the AKAR3-EV-NR. Furthermore, we found only a marginal response of the original AKAR3 sensor, showing that the AKAR3-EV sensor has an improved ability to visualize the phosphorylation activity of PKA and Sch9p in yeast. Cellular expression was more than sufficient, and the sensor showed a uniform distribution in cells (Fig. 1C). Of notice, the AKAR3-EV-NR sensor shows a significant increase shortly after glucose addition in the W303-1A strain. This increase is not caused by osmotic changes since 100 mM sorbitol addition did not increase FRET levels (Supplementary Fig. S2A). Conclusions about the FRET responses of AKAR3-EV directly after a transition should therefore be taken carefully. Furthermore, the AKAR3-EV shows no basal drift in FRET, which we found for AKAR3. The original AKAR3 sensor also shows a small dip in FRET response after the glucose addition in W303-1A, which could come from the differential pH sensitivity of the fluorescent proteins in this sensor. AKAR3-EV uses YPET-eCFP as a FRET pair, which shows more pH robustness compared to the eCFP-Venus FRET pair used in AKAR3 (Supplementary Fig. S2B) (Botman et al. 2019). Lastly, expression levels of AKAR3-EV did not affect basal FRET levels, and growth was not affected on various carbon sources (Supplementary Figs. S2C and S2D). In conclusion, the AKAR3-EV can be used in yeast to assess cellular phosphorylation of the RRxT motif.

### AKAR3-EV responses are dependent on sch9 and PKA signalling, but not ypk1

**Figure 2.**
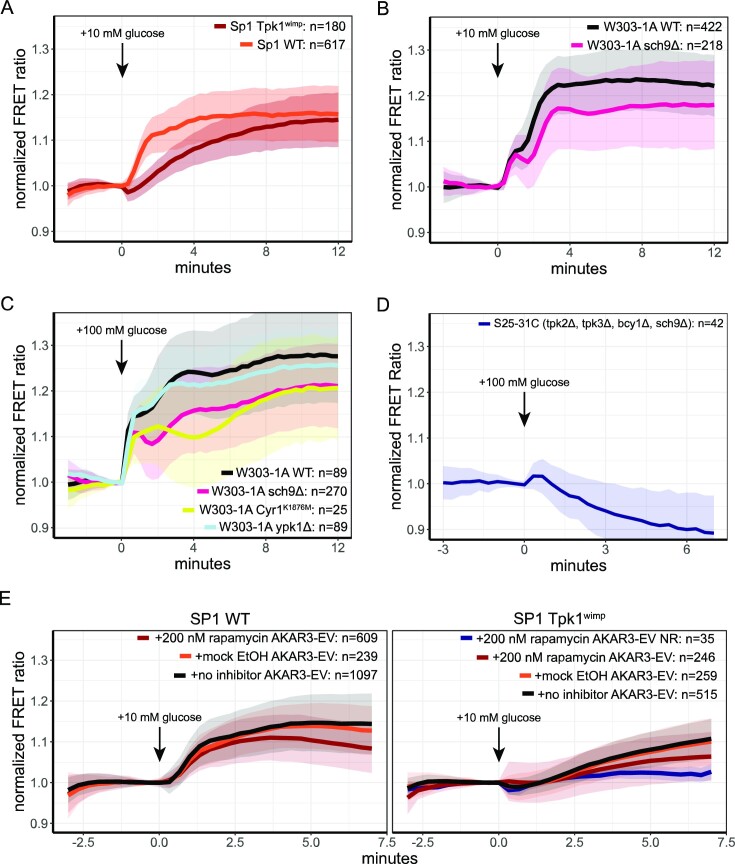
AKAR3EV responds to both Sch9 and PKA activity. (A) FRET response of SP1 WT and SP1 TPK1^wimp^ cells expressing AKAR3EV grown on 1% ethanol to 10 mM glucose addition. (B) FRET response W303-1A WT and W303-1A sch9Δ cells expressing AKAR3EV grown on 1% ethanol to 10 mM glucose addition. (C) FRET response W303-1A WT, W303-1A sch9Δ, W303-1A CYR1^K1876M^, or W303-1A ypk1Δ cells expressing AKAR3EV grown on 1% ethanol to 100 mM glucose addition. (D) FRET response of S25-31C cells expressing AKAR3EV grown on 1% ethanol to a 100 mM glucose pulse. (E) FRET response of SP1 WT and SP1 TPK1^wimp^ cells expressing AKAR3EV or AKAR3-EV NR to 10 mM glucose addition. Cells were grown on 1% ethanol, incubated for at least 2 h with rapamycin, mock (ethanol), or without any addition and pulsed with 10 mM glucose. Lines show the mean FRET value, normalized to the baseline, shades indicate SD, colours indicate the expressed sensor, strain, and/or treatment. All data are obtained from at least two biological replicates.

To assess the whether the AKAR3-EV sensor is influenced by cAMP-PKA and TORC1-Sch9 signalling, we compared normalized FRET responses of W303-1A and SP1 WT to strains carrying the *TPK1^wimp^* mutation (which has low PKA activity) and the *sch9∆* strain upon a shift from 1% ethanol to 10 mM glucose (Figs. 2A, B and Supplementary Figs. S3A, S3B). We also included non-normalized FRET responses in figure S3, as the normalized FRET responses do not show the starting phosphorylation status of a cell but only report relative changes. The *TPK1^wimp^* shows a lower initial response but does gradually obtained a similar change in normalized FRET at the end of the timelapse (normalized FRET levels of 1.16 and 1.14 for WT and *TPK1^wimp^*, respectively). In contrast, *sch9* deletion gave a reduction in the reached plateau of 25% (normalized FRET levels of 1.24 and 1.18 for WT and *sch9Δ*, respectively). To note, for both the TPK1^wimp^ and the sch9Δ, we found increased absolute FRET levels during the complete transition, indicative for a higher phosphorylation state. We also tested whether W303-1A CYR1^K1876M^, which lacks the classical cAMP peak upon glucose addition shows an altered AKAR3-EV response. This strain showed a normalized FRET response that reached a plateau similar to the sch9Δ strain (Figs. 2C and Supplementary Fig. S2C), but with different dynamics. Its absolute FRET levels were comparable to WT (Supplementary Fig. S3C). Finally, the RRxT motif can potentially also be phosphorylated by YPK1p and YPK2p (Chen et al. 1993) with YPK1p being the dominant protein. This kinase is regulated by TORC2, and this signalling branch is involved in lipid metabolism and can potentially also be activated by sugars (Jacquier and Schneiter 2010, Plank et al. 2020). We tested the FRET response of AKAR3-EV in a W303-1A *ypk1Δ* strain and found a negligible decrease in the maximal response (1.28 versus 1.26 normalized FRET levels for WT and *ypk1Δ*, respectively, Fig. 2C and Supplementary Fig. S3C). Since both the cAMP-PKA and TORC1-Sch9p cascades affect the AKAR3-EV response, we assessed whether a strain mutated in both cascades showed any increase in FRET levels upon glucose addition. For this, we used the S25-31C strain (tpk2Δ, tpk3Δ, bcy1Δ, and Sch9Δ), which is known to have impaired phosphorylation activity assessed by trehalase activity (Crauwels et al. 1997). As expected, 100 mM glucose addition to this strain showed no increase in FRET levels of AKAR3-EV, indicative that AKAR3-EV FRET (and hence RRxT phosphorylation) levels rely on PKA and Sch9p signalling (Fig. 2D and Supplementary Fig. S2D). In contrast, there is a slight decrease in FRET signal, which may be explained by glucose-induced phosphatase activity on the RRxT motif. The phosphorylation state of the sensor is in the end a steady-state balance between phosphorylation and dephosphorylation activity.

Finally, we confirmed the dependency of RRxT phosphorylation on Sch9p and PKA by the simultaneous suppression of both pathways. For this, we used the TORC1 inhibitor rapamycin to inhibit this signalling cascade and combine this with the SP1 WT and *TPK1^wimp^* strains, which have decreased (but not completely absent) PKA activity. In wild-type SP1, we found a decrease of 27% in the FRET response of rapamycin-treated cells compared to untreated cells after a 10 mM glucose pulse (Fig. 2E and Supplementary Fig. S2E). SP1 *TPK1^wimp^* cells already showed a 27% decreased maximal response (compared to WT cells), which further decreased to 60% when treated with rapamycin. This response was still higher compared to the response of the non-responsive sensor, which most likely is attributed to the remaining activity of *TPK1^wimp^*.

In conclusion, we show that the AKAR3-EV sensor can be used to measure TORC1-Sch9p and cAMP-PKA activity by visualizing the cellular RRxT phosphorylation status. Deletion of *SCH9* or removal of the classical cAMP peak results in a decreased phosphorylation status of the cell directly after the carbon source transition. In contrast, decreased PKA activity gives a decreased initial response, but eventually reaches the same plateau as wild-type cells within the short timeframe measured. Thus, the individual influence of Sch9p and PKA on the phosphorylation dynamics can be further elucidated by the AKAR3-EV sensor.

### Sugar transitions shows distinct phosphorylation dynamics with single-cell heterogeneity

**Figure 3.**
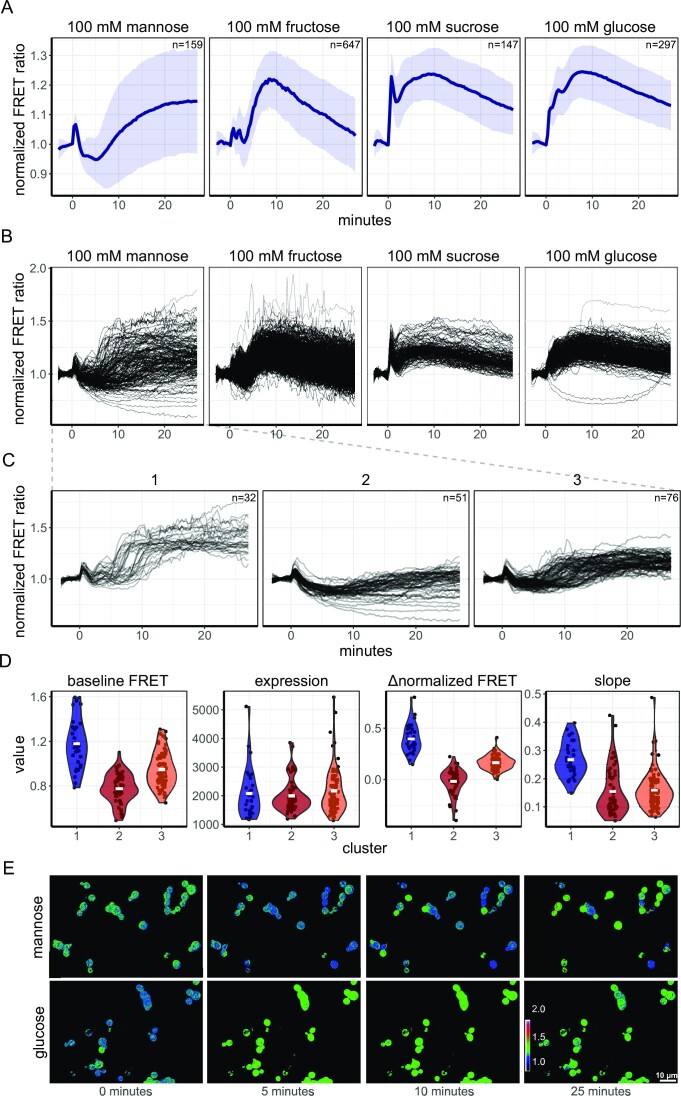
Single-cell assessment of AKAR3EV responses shows heterogeneity for mannose, but not for fructose, sucrose, and glucose additions. (A) Response of W303-1A WT cells expressing AKAR3EV grown on 1% ethanol to the various sugar additions, added at *t* = 0 minutes. Lines show mean FRET value, normalized to the baseline, shades indicate SD. Facet titles show the final concentration of the added sugar. (B) Baseline-normalized single-cell traces of the transitions depicted in A. (C) The three identified clusters found for the heterogenic response after 100 mM mannose addition. Lines depict single-cell traces of the normalized FRET value. (D) Violin plots of basal FRET level (not baseline normalized), the sum of the donor and acceptor emission fluorescence as a proxy for sensor expression, the change in normalized FRET values after 50 minutes (Δnormalized FRET and the maximal slope obtained (normalized FRET change per minute). Each dot depicts a single cell, horizontal bar presents the mean. All data are obtained from at least two biological replicates. (E) Static pictures of a 1% ethanol to 100 mM mannose or 100 mM glucose transition. Colours indicate FRET ratio (normalized to the first baseline frame), mannose was added at 0 minutes. Scale bar indicates 10 µm.

In our previous study, we found that cAMP signalling in yeast is different for different sugars, and it was homogeneous with no clear subpopulations or non-responders (Botman et al. 2021). We used the AKAR3-EV sensor to measure the downstream response to different sugars. W303-1A cells grown on 1% ethanol medium were pulsed with either 100 mM glucose or sucrose (both Gpr1p agonists), 100 mM fructose (no Gpr1p agonist or antagonist), or 100 mM mannose (an antagonist of Gpr1p) (Lemaire et al. 2004, Botman et al. 2021). Glucose, sucrose, and fructose gave a clear transient increase in the FRET ratio after addition, where sucrose and glucose gave a slightly faster and more sustained response. In contrast, mannose showed a decline in FRET signal after which the FRET response increased to higher levels, which was sustained until the end of the timelapse recording (Fig. 3A, Supplementary Fig. S4 for SP1 responses). For fructose, glucose, and sucrose, we found no clear and significant subpopulations. The most striking response at the single-cell level, however, was found for mannose addition. Mannose addition gave a highly heterogenous response (Figs 3B–E, Supplementary movie S1), which was not found for the non-responsive sensor (Supplementary Fig. S5) and was not caused by sensor expression levels, as the sum of the donor and acceptor emission (used as a proxy for sensor expression) was similar among the clusters (Fig. 3D, second panel). After the dip in FRET levels (which all cells do seem to have), we identified three discrete responses, which we clustered using k-means clustering. The first cluster consisted of cells that have a broad timeframe in which cells increase in FRET levels. Furthermore, final FRET levels are slightly increased (mean normalized FRET value of 1.08), and the slope of the FRET increase was high (mean normalized FRET increase of 0.26 per minute). The second cluster showed clear switchers, which obtained a final normalized FRET value of 1.25. Finally, the third cluster consisted of cells that did not increase but decreased their RRxT phosphorylation levels (a mean normalized FRET value of 0.90 at the end of the timeframe). Interestingly, for the mannose transitions, cells that had a lower baseline FRET value also seem to end in a lower FRET state, and vice versa (Fig. 3D).

**Figure 4.**
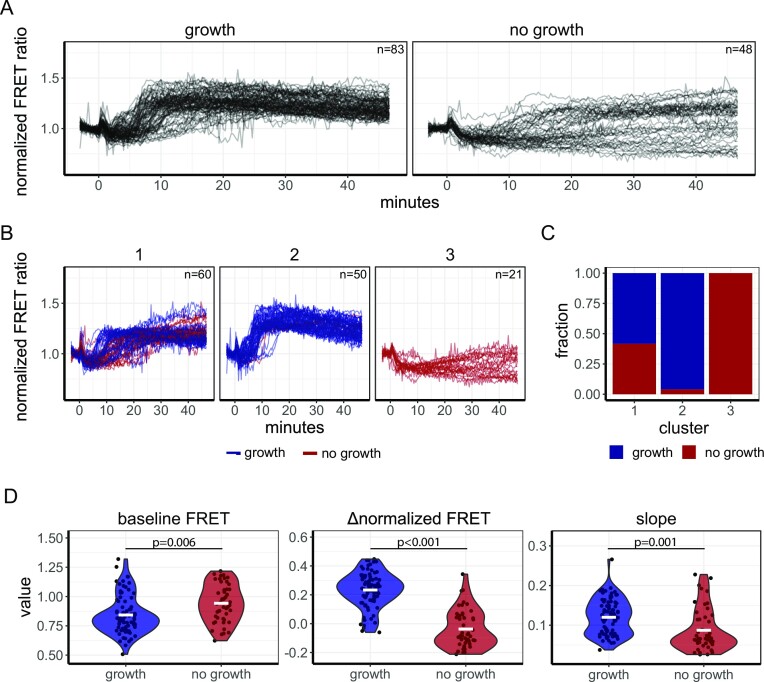
AKAR3-EV shows differential FRET responses based on the growth state upon mannose addition. (A) Response of W303-1A WT cells expressing AKAR3EV grown on 1% ethanol to 100 mM mannose, added at *t* = 0 minutes. Lines show the normalized FRET value per cell. Facet titles show whether the cell was a confirmed grower or a non- grower. (B) The three identified clusters found after 100 mM mannose addition. Lines depict single-cell traces of the normalized FRET value, colours depict whether cells grow or do not grow (C) Histogram depicting which fractions of cells grow (blue) or not grow (red) for each cluster. (D) Violin plot of the basal FRET level (not baseline normalized), the change in normalized FRET values at the end of the recorded timelapse (Δnormalized FRET), and the maximal slope obtained (normalized FRET change per minute). Each dot depicts a single cell. White bar represents the mean. *P* values from student’s *t*-test are shown. All data obtained from three biological replicates.

We further explored the heterogeneous FRET response after mannose addition by evaluating the cellular growth of these yeast for at least 2 h after mannose addition. For accurate growth assessment, analysis was restricted to cells that had a bud at the start of the experiment or developed a bud during the experiment. (Fig. 4). Single-cell FRET curves were also determined (Fig. 4A), and k-means clustering was performed (Fig. 4B). We found again the 3 clusters, and when we overlayed growth versus non-growth in these clusters (Fig. 4B and C), we found one cluster with mostly growers, one cluster with non-growers, and a mixed cluster. Growing cells had a significant lower basal FRET level (0.84 ± 0.16 versus 0.94 ± 0.16 for growing and non-growing, respectively. Student’s *t*-test, *P* = 0.006, Fig. 4D), obtained a larger normalized FRET change (0.20 ± 0.09 versus 0.07 ± 0.20 for growing and non-growing, respectively. Student’s *t*-test, *P* < 0.001, Fig. 4D), and showed a faster normalized FRET increase per minute (0.12 ± 0.04 versus 0.09 ± 0.05, *P* = 0.001).

In conclusion, the AKAR3-EV sensor shows multifarious responses after sugar addition, dependent on the specific sugar. For mannose, we found a highly heterogenous response, and its clustering suggests that the rapid dynamics of the RRxT phosphorylation status during a carbon-source transition correlates with the onset of rapid growth.

### The nutrient-induced phosphorylation system has a high affinity for glucose

**Figure 5.**
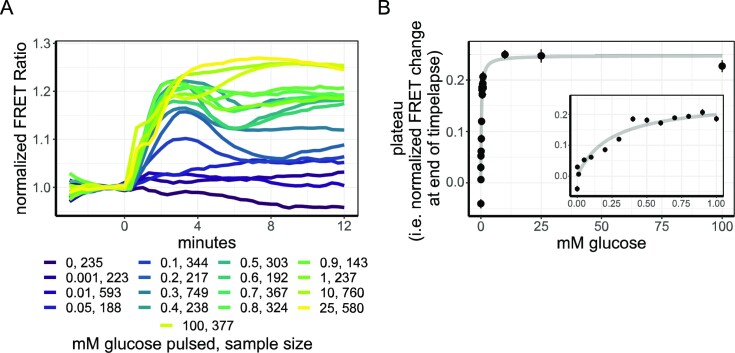
Dose-response of AKAR3-EV to glucose addition shows a high affinity. (A) dose-response curves of 1% ethanol-grown W303-1A cells expressing AKAR3-EV to various concentrations of glucose additions, added at *t* = 0 minutes. Lines show the mean baseline-normalized FRET response. Colours indicate the final glucose concentration after glucose addition. (B) Final FRET response of AKAR3-EV-expressing cells to the glucose concentration added, dots show mean response, error bars indicate SD, and grey line indicates fit (from equation 1). Fitting of the final AKAR3-EV FRET levels to the glucose concentration pulsed shows saturation kinetics with a K_0.5_ of 0.24 and a maximal normalized FRET value of 1.24. Inset shows the zoom-in of the AKAR3-EV responses from 0 to 1 mM of glucose additions. All data obtained from at least two biological replicate.

When we tested the response of the AKAR3-EV sensor in W303-1A to different levels of glucose (Fig. 5A), we found a graded response that could be fitted by a single binding curve with a relatively high affinity, with a K_0.5_ of 0.27 mM for glucose (Fig. 5B). A dose-response curve of strain SP1 gave similar parameters (K_0.5_ of 0.26 mM), indicating that this high affinity is not a strain-specific feature (Supplementary Fig. S6). In addition, no clear subpopulations or non-responders were detected (Supplementary Fig. S7). This is in line with previous results found for cAMP (Botman et al. 2021).

In conclusion, we found that the affinity of the RRxT phosphorylation system (i.e. TORC1-Sch9p and cAMP-PKA) has a high affinity for glucose—in comparison, the affinity we found for cAMP peak height was 3.0 mM, ten times higher, therefore. Furthermore, this system appears to be homogeneous since no clear non-responders or subpopulations were found.

### AKAR3-EV shows no growth-rate dependent FRET status

**Figure 6.**
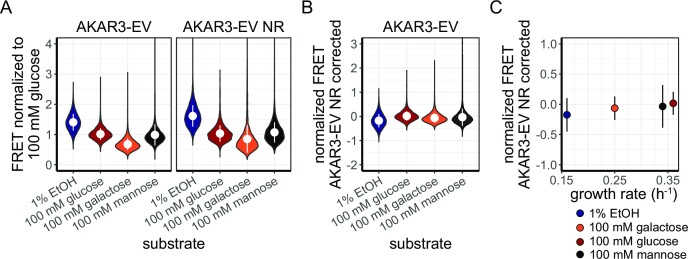
Growth rate does not relate with AKAR3EV basal levels. (A) Violin plot of basal AKAR3EV and AKAR3EV NR FRET levels measured using flow cytometry in W303-1A WT on various carbon sources, normalized to the 100 mM glucose condition. White dot depicts median FRET value, errorbars indicate SD. (B) Violin plot of AKAR3-EV FRET levels after correcting for aspecific signal on the four carbon sources. White dot depicts the median FRET value, error bars indicate SD. (C) AKAR3-EV FRET levels (corrected by AKAR3-EV NR signal) plotted against the growth rate of W303-1A WT on the carbon sources. Each dot depicts the population average FRET level on a specific carbon source (indicates by the dot colour), error bars indicate SD. Representative dataset shown from two biological replicates.

After characterizing the TORC1-Sch9p and cAMP-PKA signalling response during nutrient transitions, we studied the steady state phosphorylation levels during (balanced) growth on different carbon sources. W303-1A cells expressing either the AKAR3-EV or the AKAR3-EV-NR sensor were grown to mid-exponential phase on 1% ethanol (growth rate of 0.16 h^−1^), galactose (growth rate of 0.25 h^−1^), glucose (growth rate of 0.36 h^−1^), or mannose (growth rate of 0.34 h^−1^), and the FRET level distributions across a population were measured using a flow cytometer (Fig. 6A). We found significant differences between the conditions tested (Kruskal–Wallis, *P* < 0.01), except between galactose and mannose (Wilcoxon signed-rank test, *P* = 0.6). However, we also found such differences in the non-responsive sensor, and no clear relation was found between growth rate and the FRET levels of AKAR3-EV after correction for aspecificity (Figs. 6B and C).

## Discussion

In the present study, we implemented the mammalian-optimized AKAR3-EV sensor in yeast and tested whether this sensor can also be applied to study Sch9p and PKA signalling in yeast. In yeast, the sensor has sufficient expression and a homogenous distribution in cells. Furthermore, growth rates were not affected by the sensor, indicating the sensor is harmless to cells (Supplementary Fig. S1C). The AKAR3-EV sensor showed the expected response to an ethanol-to-glucose transition and outperformed a previously published AKAR sensor in yeast (Fig. 1A). The baseline drift of the original AKAR sensor can be caused by the photochromic behaviour of one of the FPs (Botman et al. 2019), or differential FP responses to changes in the intracellular composition. We also made a non-responsive version of AKAR3-EV by mutating the RRxT motif to RRXA (Plank et al. 2022). This AKAR3-EV NR sensor indeed showed significantly lower responses, although we found that some transitions evoked a rather large response immediately after the transition. The origin of this response is not known and may be caused by nonspecific phosphorylation of the sensor domain, although this domain does not contain any other known phosphorylation sites of PKA or Sch9p. This response can also originate from changes of the intracellular composition (e.g. changes in redox potential, pH, and ion concentration). If needed, the AKAR3-EV NR can be used to correct for these aspecific responses. This sensor can be potentially improved for use in yeast by using phosphorylation sites that are more common for yeast (i.e. RRXS) (Plank et al. 2022), although this could also increase the basal phosphorylation status of the sensor, decreasing its dynamic FRET range. In addition, (y)mTurquoise2 could be used instead of eCFP since this fluorescent protein is a better FRET donor (Goedhart et al. 2012, Mastop et al. 2017).

The AKAR3-EV FRET response is indeed dependent on both TORC1-Sch9p and cAMP-PKA signalling (Fig. 2). Glucose addition to ethanol-grown cells showed that impaired PKA signalling (using the *TPK1^wimp^* strain) decreased the rate of phosphorylation, although the maximal response remains similar. An *Sch9* deletion, on the other hand, resulted in a lower normalized response after a glucose transition. Yet, these transitions were performed in different strains (SP1 and W303-1A, respectively). Interestingly, we also found that the W303-1A *CYR1^K1876M^* mutation in which the transient cAMP peak is absent (Botman et al. 2021) showed a decreased maximal response. Further research should clarify whether and why missing the transient cAMP peak has a long-term effect on cell signalling status and fitness. The S25-31C strain with impaired signalling in both Sch9 as PKA, showed a decrease in RRxT phosphorylation, proving that the AKAR3-EV sensor indeed measures solely PKA and Sch9p activity. This was further confirmed by adding rapamycin in SP1 WT and SP1 TPK1^wimp^ cells, where the rapamycin-treated TPK1^wimp^ strain showed a severe reduction in FRET response after glucose addition. Lastly, we found no significant effect of *ypk1* deletion on the FRET response of AKAR3-EV in W303-1A, indicating that the AKAR3-EV sensor measures specifically PKA and Sch9p activity. As mentioned, some mutant strains had higher absolute FRET levels, suggesting that the signalling architecture can compensate for a decreased kinase activity. This can occur, for example, via negative feedback patterns known for PKA (Nikawa et al. 1987, Nikawa et al. 1987, Dong and Bai 2011) and TORC1 (Péli-Gulli et al. 2017) or altered activity of RRxT phosphatases. Another possibility is that an altered cellular composition confounds FRET levels (Moussa et al. 2014), for which the non-responsive sensor can function as a control. Thus, as for every FRET sensor, quantitative conclusions about normalized and absolute FRET levels should be taken with care. In this study, we did not wish to make quantitive conclusions about signalling mutants on the absolute phosphorylation status, but rather use these mutants to show that the AKAR3-EV sensor indeed measures the activity of these two kinases.

A major strength of biosensors is the ability to measure single-cell responses. Therefore, we assessed single-cell responses during transitions from ethanol-grown cells to glucose, sucrose, fructose, and mannose (Fig. 3). As previously found for cAMP signalling (Botman et al. 2021), hardly any heterogeneity or subpopulations were found for the glucose, sucrose, and fructose transitions. In contrast, we did find a heterogenic response upon mannose addition. Mannose is known as an antagonist of cAMP signalling (Lemaire et al. 2004, Botman et al. 2021), but is metabolized at rates similar to glucose as cells grow comparable on these sugars (growth rate of 0.34 h^−1^ for mannose and 0.36 h^−1^ for glucose). Glycolytic startup, determined by pH measurements, also indicates that mannose is transported and metabolized at least as fast as glucose (Supplementary Fig. S8). The conflicting signals between the signalling and metabolism of mannose may be the reason for the heterogenic response. Cells obtaining a higher RRxT phosphorylation state, compared to the pre-transition state, seem to start growth whereas cells with a (s)lower response seem to halt growth. This confirms that PKA and Sch9p activity, and the RRxT phosphatases have an effect on the cellular decision to start growth, in line with previous studies showing that cAMP-PKA signalling and Sch9p are involved in cell cycle progression (Hubler et al. 1993, Müller et al. 2003, Jorgensen et al. 2004, Futcher 2006, Cocklin and Goebl 2011). The heterogenous Sch9p and PKA signalling dynamics potentially transmit further downstream, where it is shown that different temporal nuclear localization patterns of transcription factors (such as msn2p, a phosphorylation target of Sch9p and PKA) can result in differential transcription and translation responses potentially resulting in an altered growth response (Hao and O'Shea 2012, Zid and O'Shea 2014, Hansen and O'Shea 2016).

The AKAR3-EV sensor revealed a relatively high affinity (K_0.5_ = 0.24 mM) of the phosphorylation system for glucose (Fig. 4 ). This is far below the affinity of the high-affinity hexose transporters in yeast with a lowest K_m_ of ∼1–2 mM, for HXT6 and 7 (Reifenberger et al. 1997, Maier et al. 2002), which are expressed in these ethanol-grown cells. Furthermore, the affinity is lower compared to our previously obtained glucose affinity of cAMP peak levels in yeast (Botman et al. 2021), which confirms that the RRxT phosphorylation status of the cell is not solely determined by cAMP-PKA signalling. The high affinity of the RRxT phosphorylation system is, on the other hand, not too far from the Monod constant for glucose-limited growth, which is around 0.5 mM, admittedly for another strain (CEN.PK) (Canelas et al. 2011). Moreover, these values are in line with previous observations for Mig1 translocation after glucose additions (Bendrioua et al. 2014). The fact that we did not find clear subpopulations at any glucose concentrations shows that, at least for glucose, yeast cells sense its concentration in a highly accurate and robust manner.

One large unanswered question is whether the basal signalling status of the Sch9p and PKA signalling pathways depends on the specific growth rate. This may be expected as transcription of ribosomal genes is an important target of the pathway, and ribosomal content does scale with growth rate (Metzl-Raz et al. 2017). The obtained flow cytometry data are statistically significantly different between most conditions, but given the small size of the difference relative to the spread of the distributions (Figs. 6B and C), their biological relevance is disputable. The small differences could be caused by a maximal phosphorylation status of the cell under the conditions tested, especially since the RRxT is a preferred motif of Sch9p and PKA (Plank et al. 2020). Yet, we showed that the FRET levels can increase for >20% in cells grown in 1% ethanol medium, and the basal FRET level of this condition is comparable with the other conditions tested. This implies that the sensor is not maximally phosphorylated. Therefore, we believe that the basal phosphorylation status of the RRxT motif is unchanged and not maximally phosphorylated between various growth rates. Unchanged RRxT phosphorylation levels do not necessarily imply that PKA and Sch9p activities are also unchanged across growth conditions, as the phosphorylation level is a result of both phosphorylation and dephosphorylation. Furthermore, PKA and Sch9p phosphorylate also other motifs, potentially with different rates across conditions (Plank et al. 2022). We hypothesize that the RRxT phosphorylation response upon sugar transitions steers cells to the right cellular physiology, after which this signalling system returns to its basal level again. The dynamics of Fig. 3A also suggest a temporal impact of sugars on the phosphorylation state.

In summary, the AKAR3-EV proved to be a robust sensor to measure the nutrient-induced RRxT phosphorylation status in yeast cells. However, since the phosphorylation status is an integrated output of the activity of both Sch9p and PKA, but also on phosphatases, its interpretation is more challenging than for other sensors. Nonetheless, we believe that the AKAR3-EV sensor is a useful addition to the toolbox that can help to elucidate how yeast cells respond and adapt to nutrient changes.

## Data and resources

AKAR3-EV and AKAR3-EV NR in pDRF1-GW can be acquired via Addgene (https://www.addgene.org/182533/and https://www.addgene.org/182534/). Data can be found via at DOI:10.17632/w655db7rj9.1

## Supplementary Material

foad029_Supplemental_Files
