# Supplementary figures and images for: Using the AKAR3-EV biosensor to assess Sch9p- and PKA-signalling in budding yeast
Source: FEMS Yeast Res. 2023 May 12;23:foad029. doi: 10.1093/femsyr/foad029 (PMC10237333; doi:10.1093/femsyr/foad029)

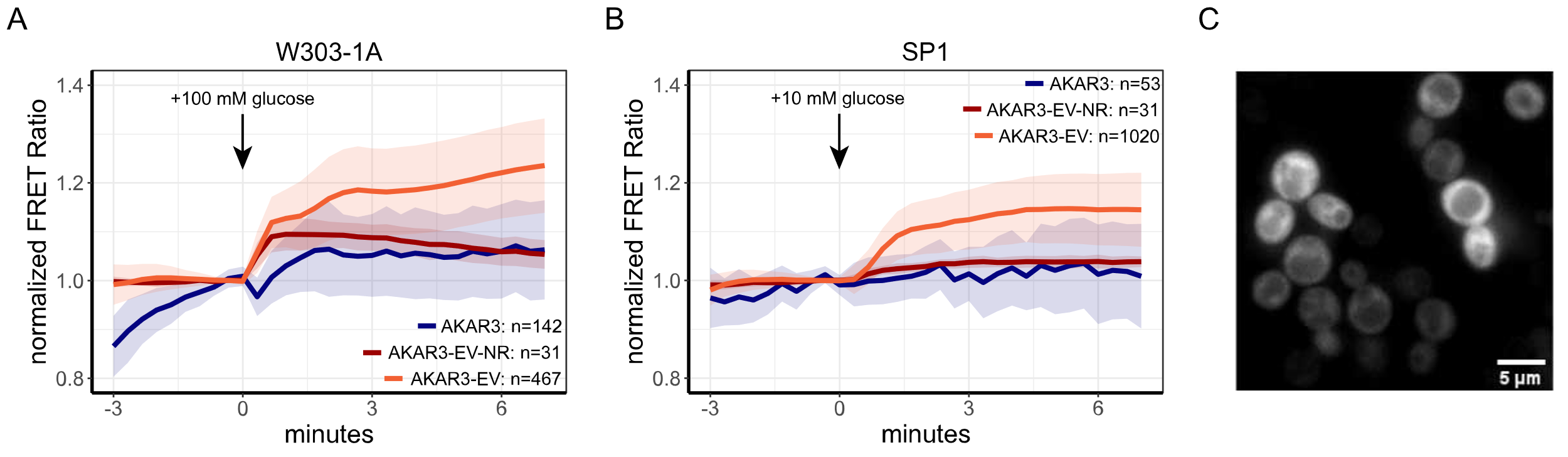


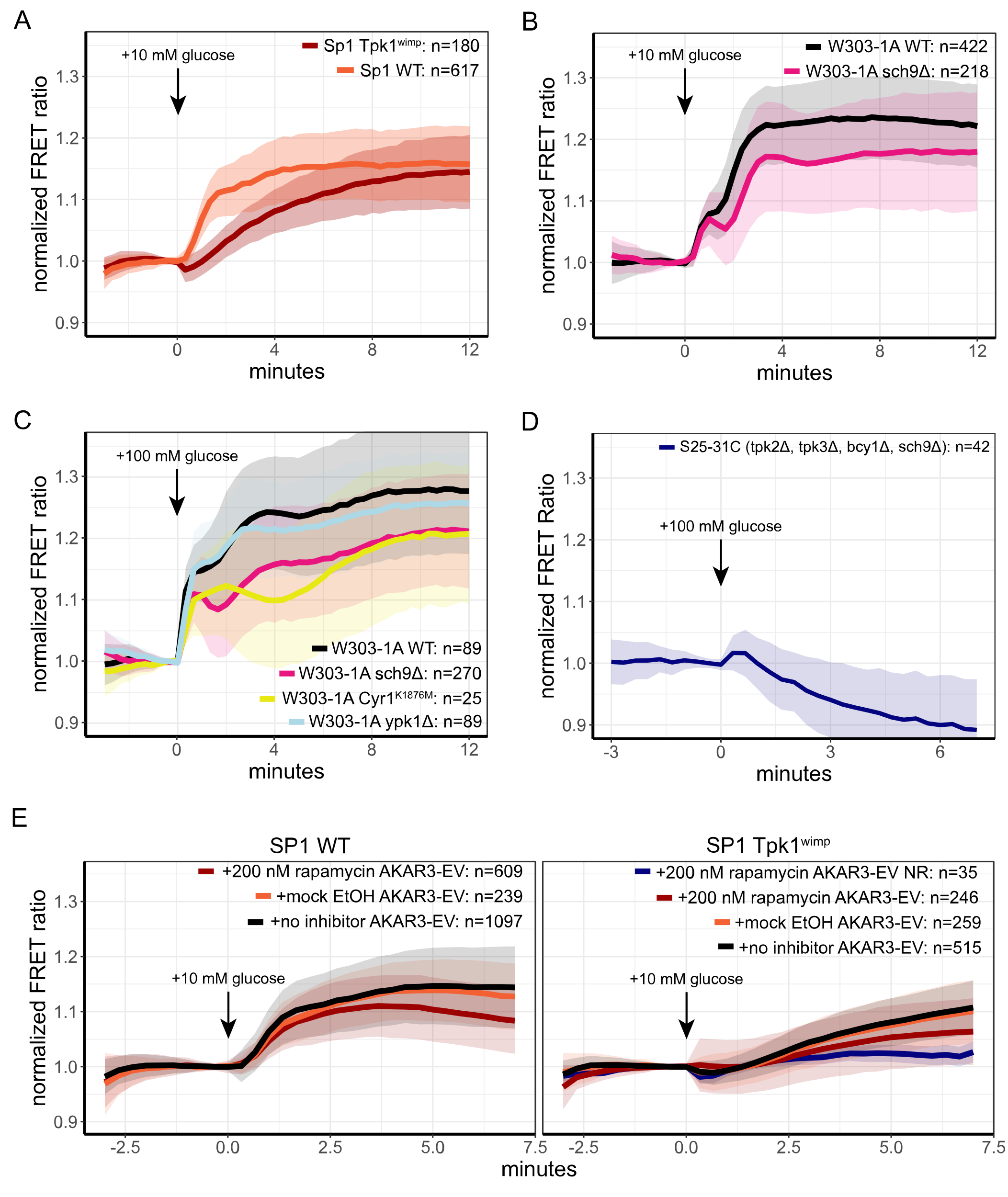


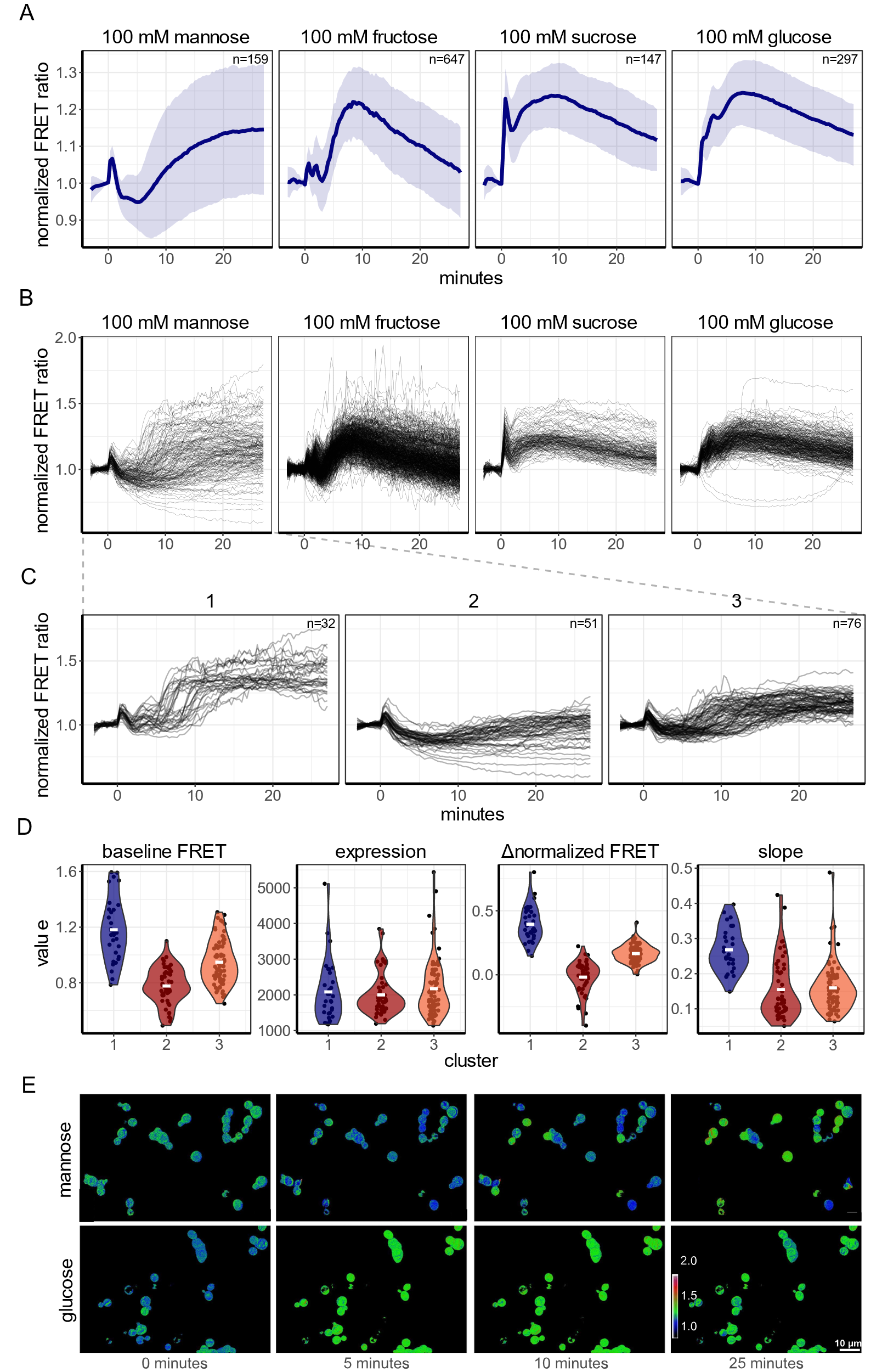


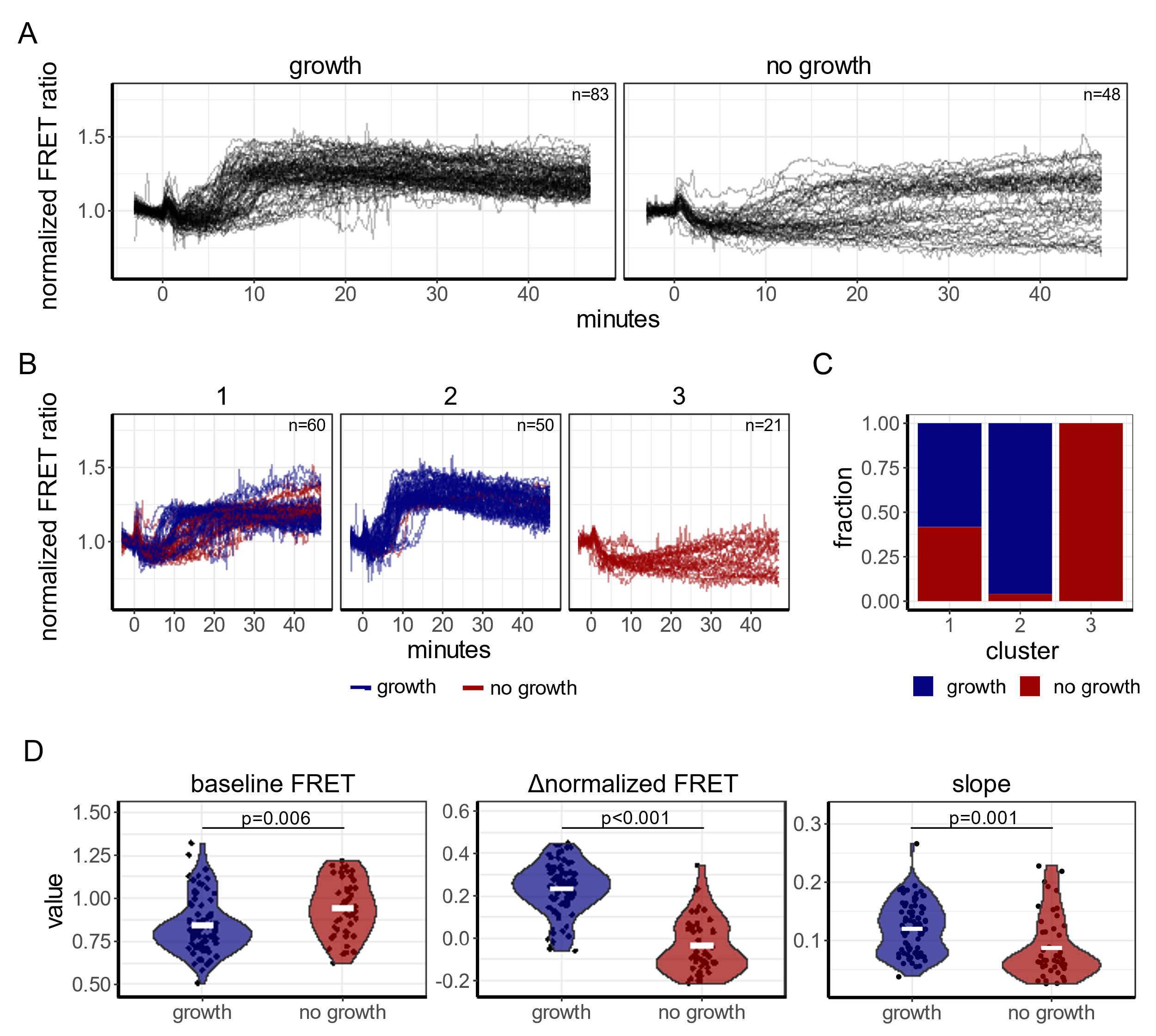


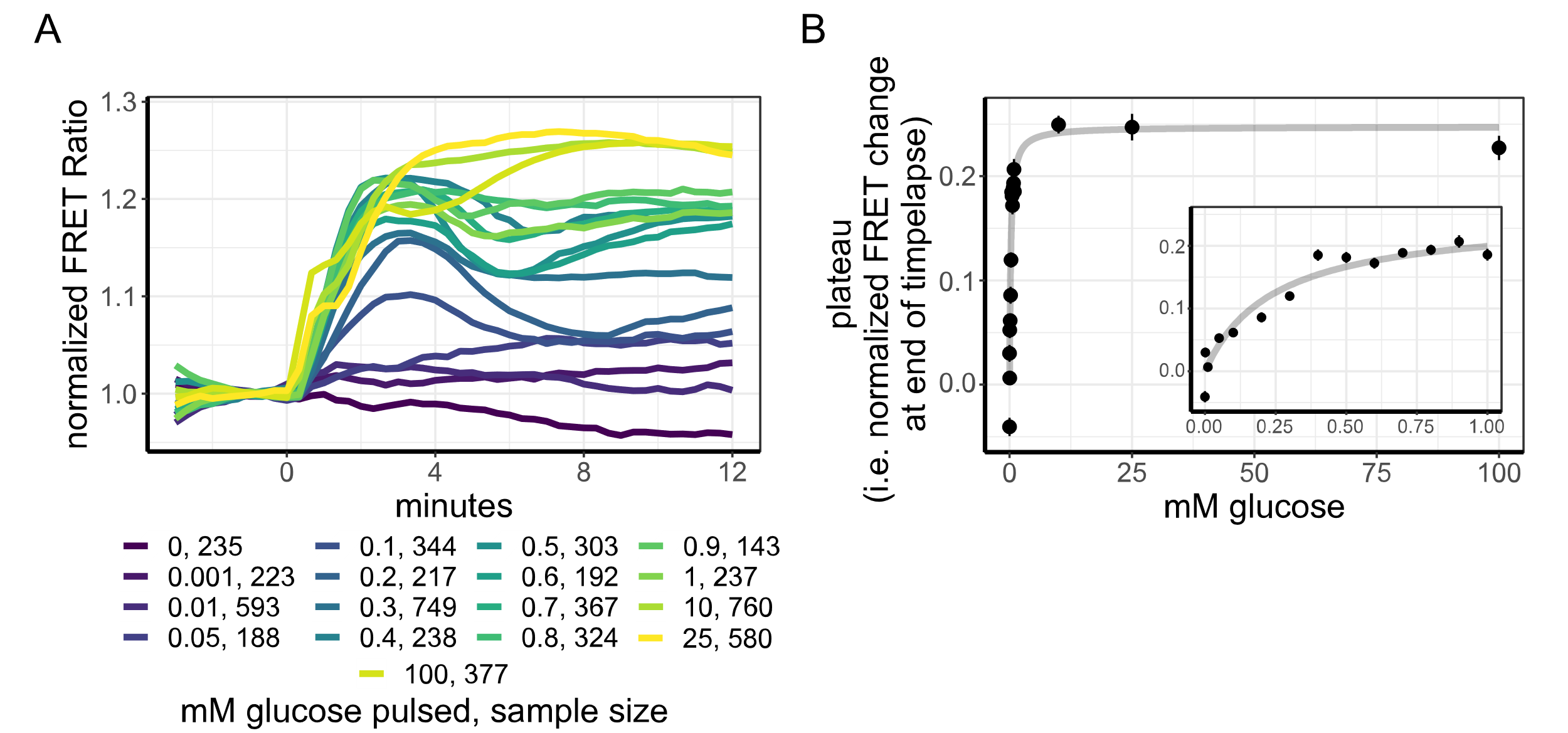


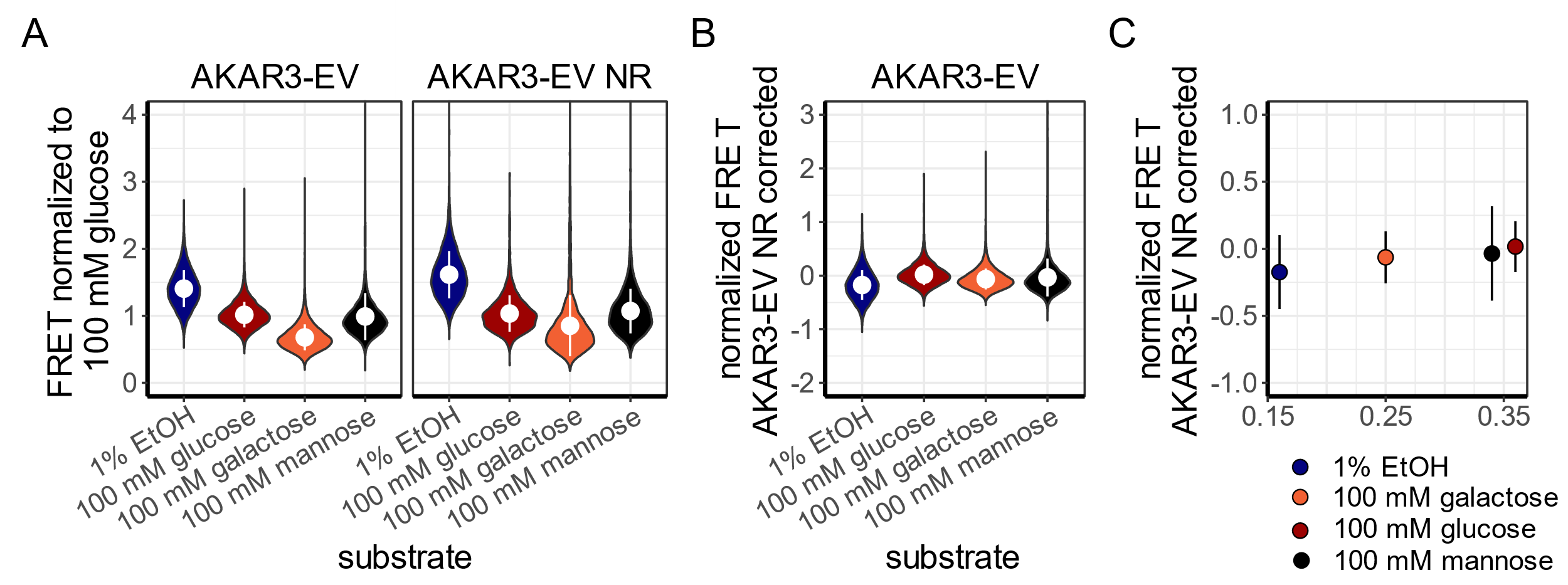

Supplement: foad029_Supplemental_Files [file foad029_supplemental_files.zip › Figures manuscript.docx]

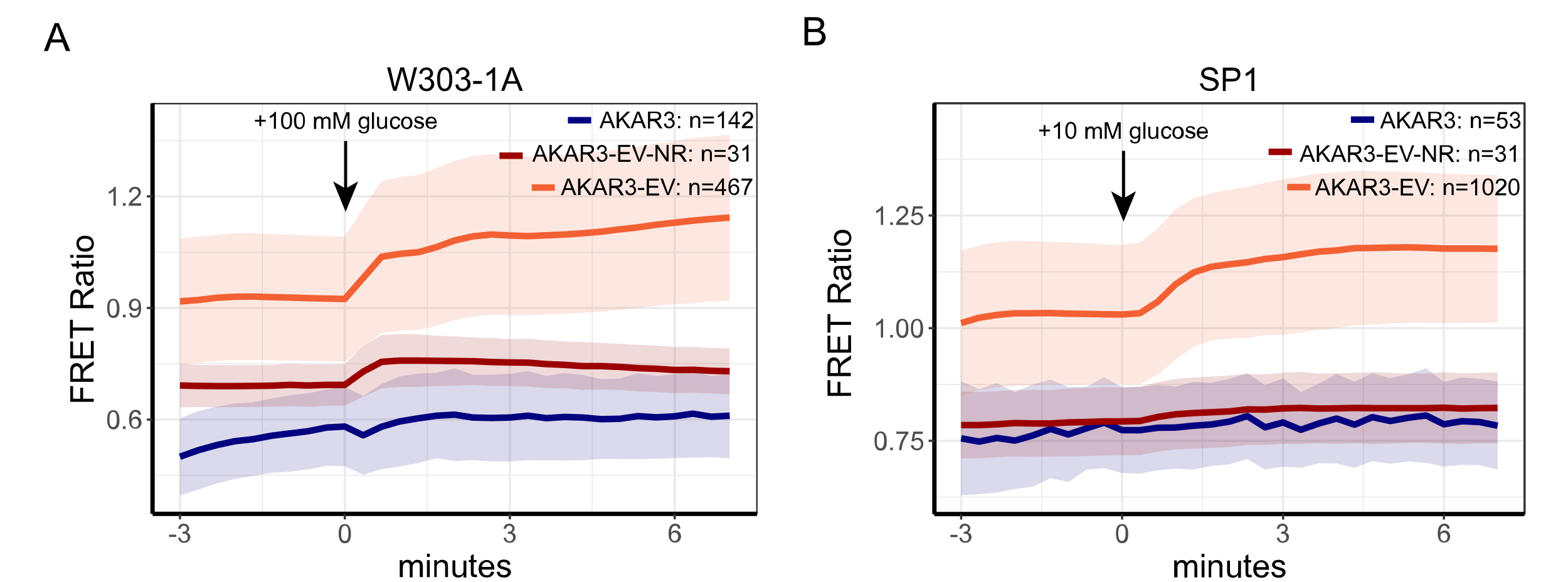


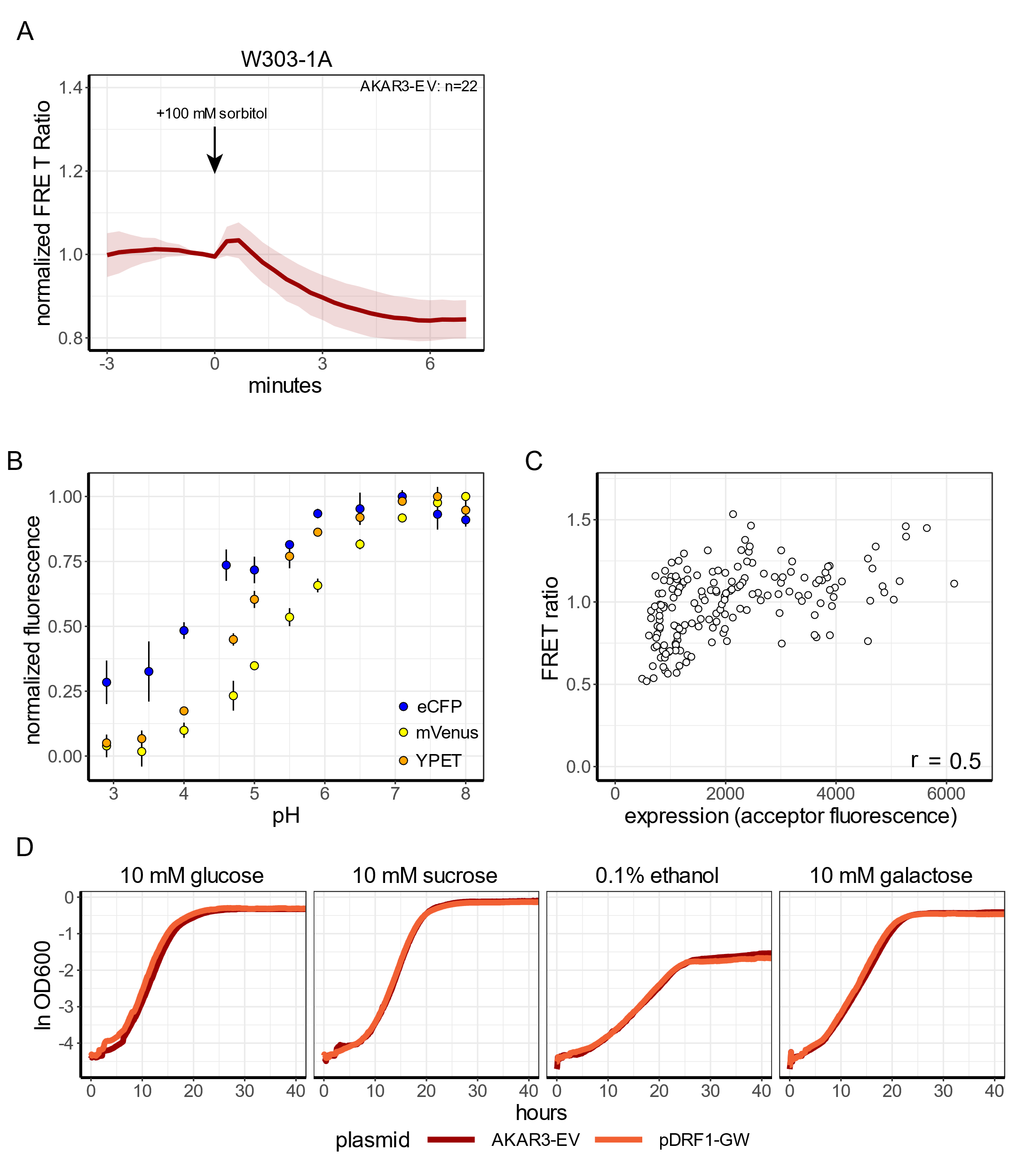


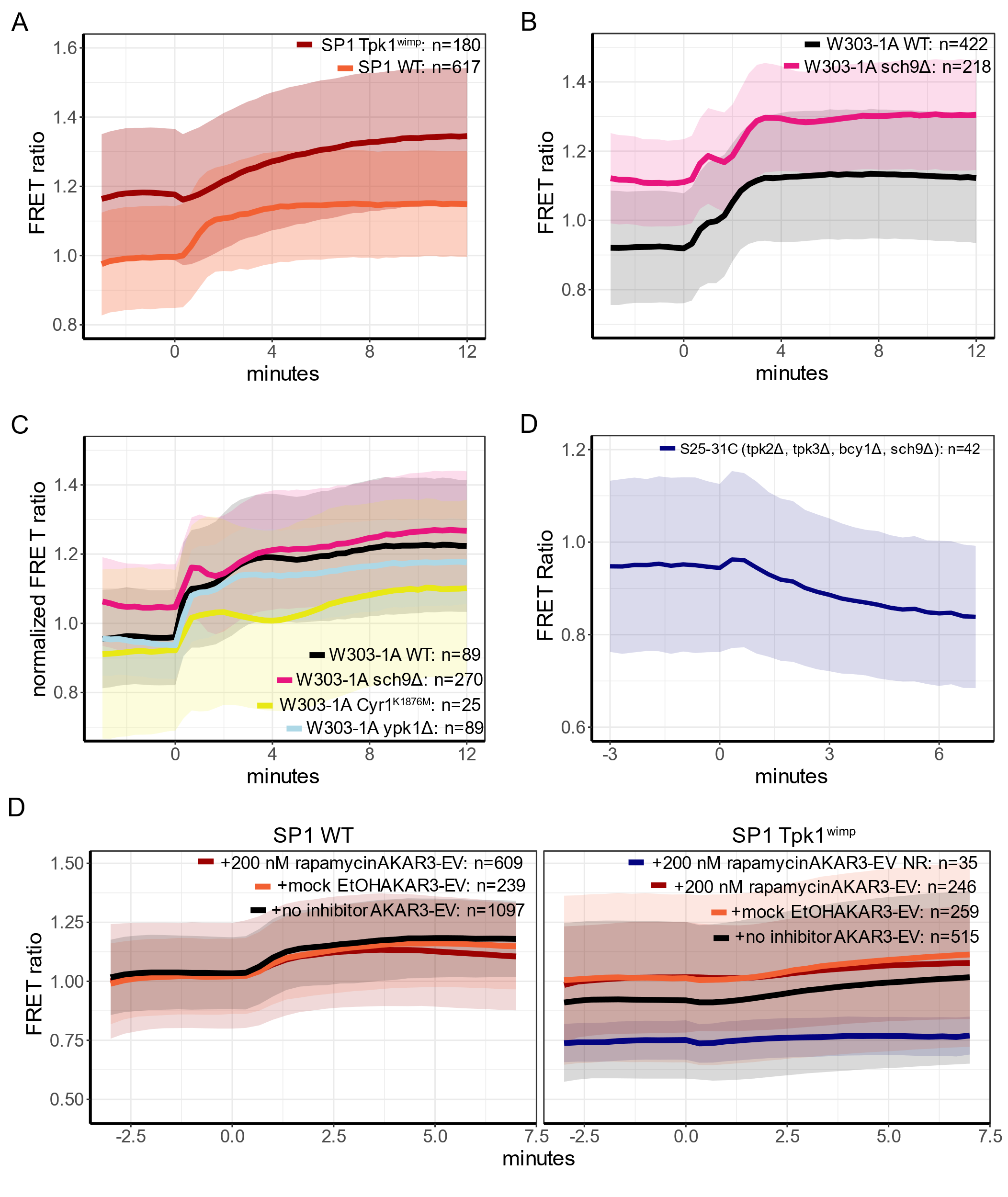


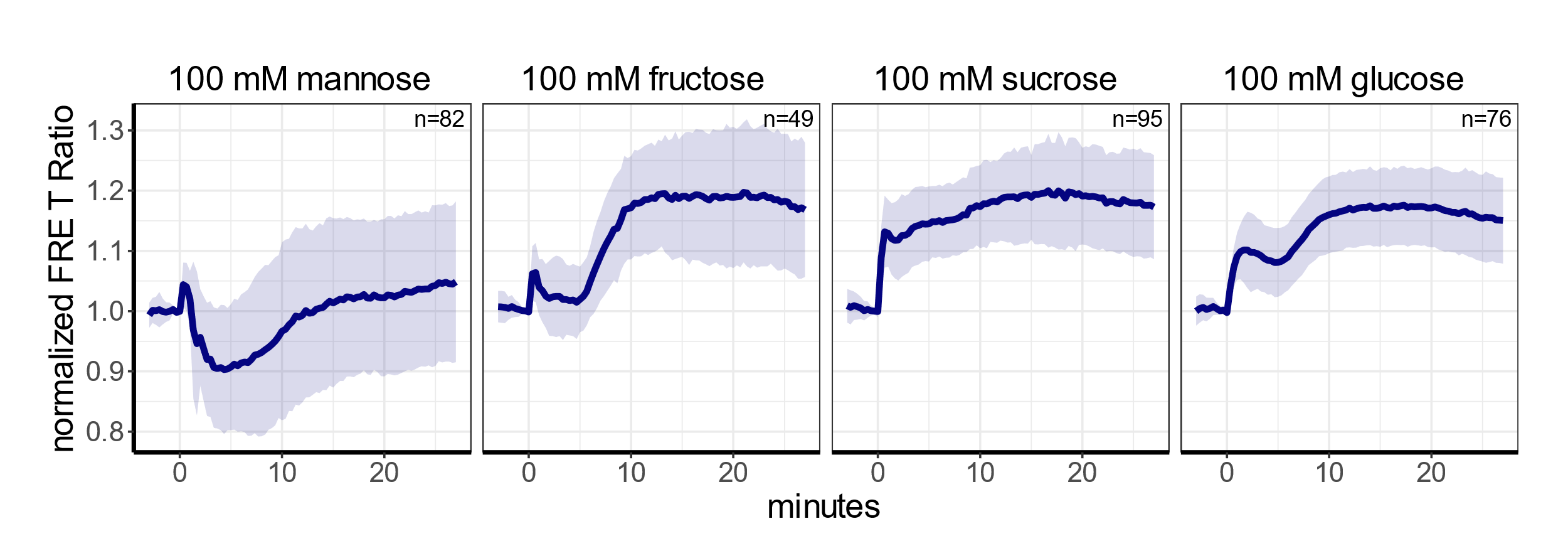


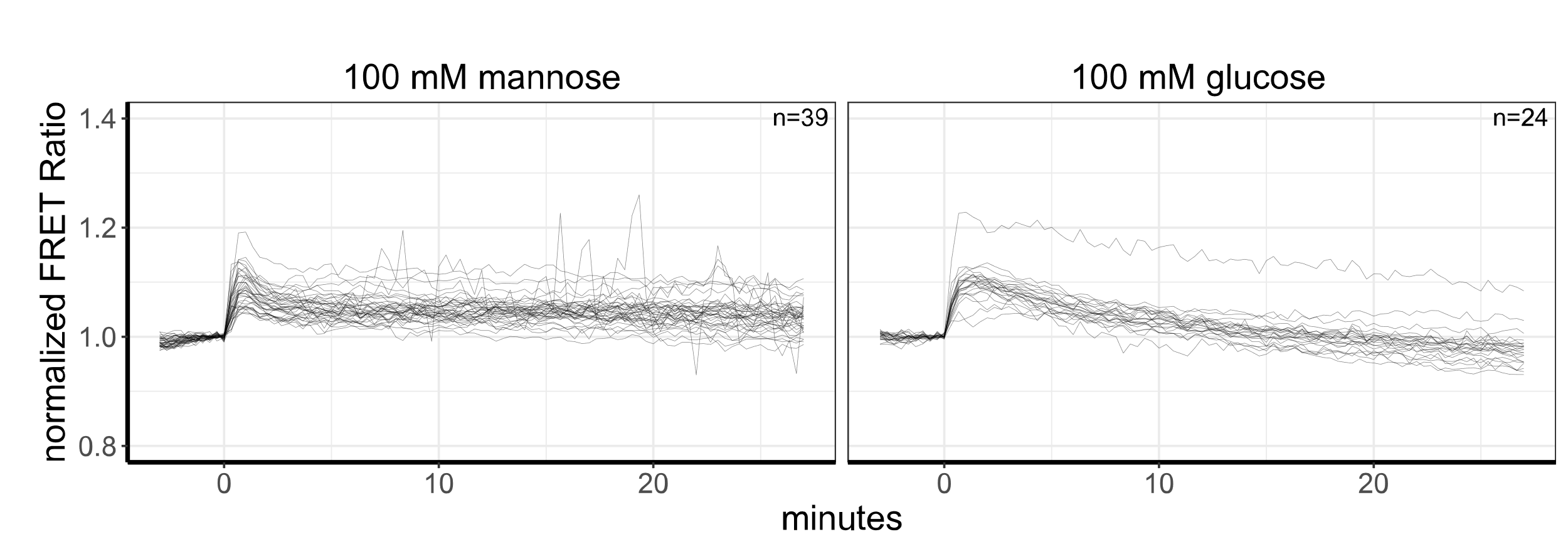


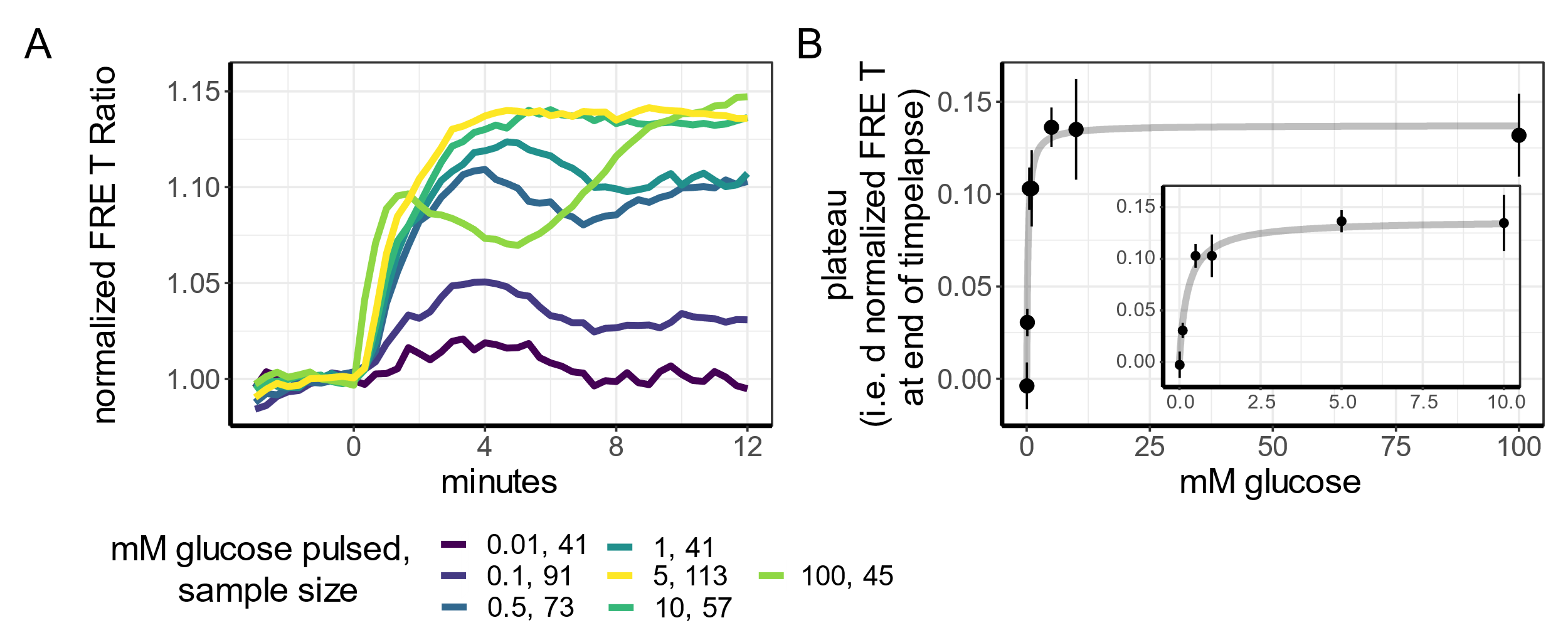


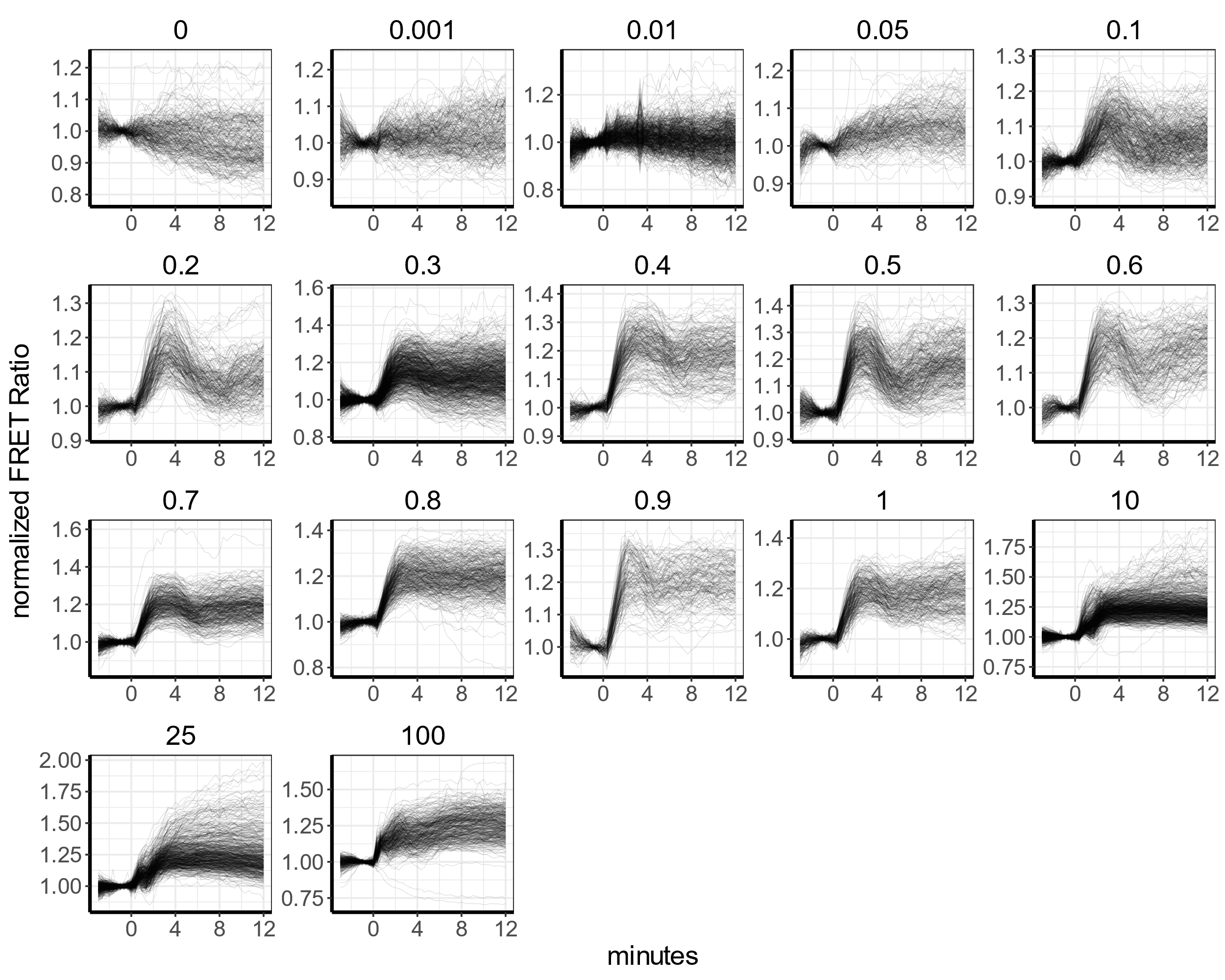


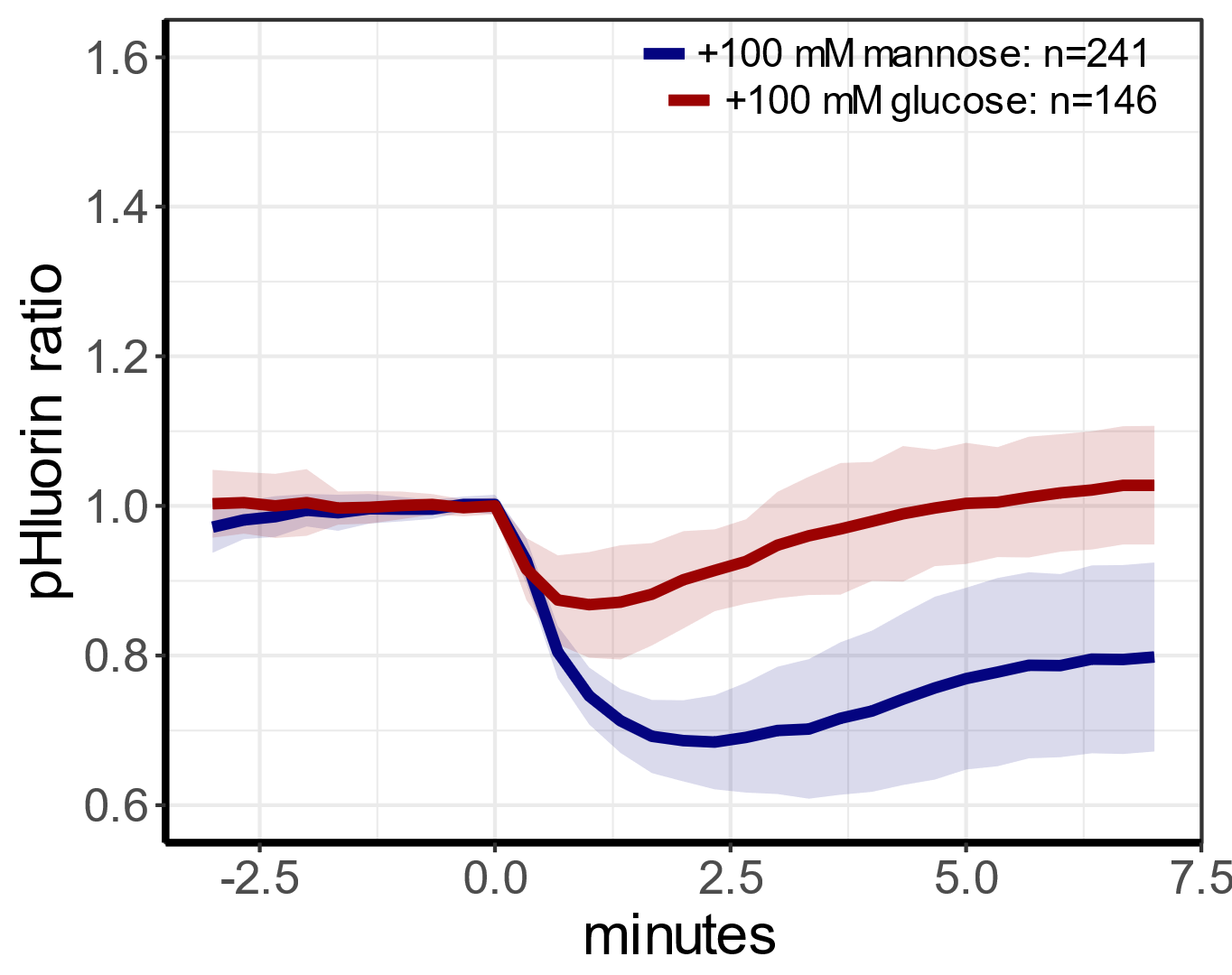

Supplement: foad029_Supplemental_Files [file foad029_supplemental_files.zip › Supplemental figures manuscript.docx]
